# Neural Control of Mastication: Ion-Channel Mechanisms in the Brainstem Central Pattern Generator

**DOI:** 10.3390/brainsci16070752

**Published:** 2026-07-15

**Authors:** Hiroki Toyoda

**Affiliations:** Department of Physiology, School of Dentistry, Aichi Gakuin University, Nagoya 464-8650, Japan; toyoda@dpc.agu.ac.jp; Tel.: +81-52-757-6741

**Keywords:** mastication, central pattern generator, ion channels, brainstem, rhythmic motor control

## Abstract

**Highlights:**

**What are the main findings?**

- Mastication is generated by a distributed brainstem central pattern generator that integrates cortical commands and sensory feedback.
- Persistent sodium, T-type calcium, HCN, and calcium-activated potassium channels contribute to the generation and modulation of masticatory rhythms.

**What are the implications of the main findings?**

- Ion-channel dynamics provide a mechanistic framework for understanding neural control of chewing.
- Targeting rhythm-related ion channels may offer novel therapeutic approaches for masticatory and orofacial disorders.

**Abstract:**

Mastication is a fundamental rhythmic motor behavior controlled by a brainstem central pattern generator (CPG) located within the pontine and medullary reticular formations. Coordinated activation of jaw-opening and jaw-closing muscles is generated by this network and continuously refined through sensory feedback from periodontal mechanoreceptors and muscle spindles, together with descending inputs from the cortical masticatory area (CMA), basal ganglia, and cerebellum. Thus, mastication is regulated by distributed neural circuits rather than a single central locus. At the cellular level, the rhythmic activity of the masticatory CPG depends on the coordinated action of voltage-gated and ligand-gated ion channels. Recent electrophysiological and computational studies have identified candidate conductances that are proposed to underlie rhythm generation. Persistent sodium currents are proposed to facilitate burst initiation, whereas T-type calcium channels are thought to promote burst activation through post-inhibitory rebound. HCN channels may contribute to rhythmic timing, while calcium-activated potassium channels are thought to participate in burst termination. This review summarizes the hierarchical neural control of mastication and the biophysical mechanisms by which ion channels shape CPG rhythmogenesis. It also discusses the impact of channelopathies and neurodegenerative disorders on masticatory function, highlighting potential ion-channel-targeted therapeutic approaches for temporomandibular disorders, bruxism, and impaired mastication.

## 1. Introduction

Mastication constitutes the initial, vital phase of the digestive process in mammals, serving to mechanically reduce solid food aggregates into a compliant bolus suitable for deglutition [1]. Far exceeding a simple mechanical crushing mechanism, chewing involves an exquisite coordination of the mandible, tongue, lips, and buccal musculature. This behavior ensures precise intraoral food manipulation, triggers salivary enzyme secretion, and facilitates the release of sapid molecules to stimulate gustatory and olfactory receptors. Mastication should be increasingly viewed as part of a broader oral multisensory network involving proprioceptive, gustatory, tactile, and nociceptive integration within distributed brain circuits [2].

Over the past two decades, neurobiological investigations have revealed that the physiological significance of mastication extends substantially beyond homeostatic nutritional processing. Rhythmic masticatory activity exerts a powerful modulatory influence on central nervous system (CNS) structures. Clinical and experimental rodent models have robustly demonstrated that chronic masticatory dysfunction—induced by tooth loss, soft-diet feeding, or occlusal disharmony—correlates directly with accelerated cognitive decline, spatial memory impairments, and structural deficits within the hippocampus [3,4,5,6]. Repetitive chewing acts as a potent stimulator of cerebral blood flow [7] and has been associated with increased BDNF/TrkB signaling in the dentate gyrus [4,8]. Furthermore, reduced masticatory stimuli during development or aging attenuate synaptic density in the CA1 and dentate gyrus regions of the hippocampus while simultaneously altering hypothalamic–pituitary–adrenal (HPA) axis activity, leading to heightened chronic stress responses [3,4]. Consequently, the preservation of optimal masticatory efficiency is increasingly recognized as a key determinant of healthy neurological aging and cognitive longevity.

While the volitional initiation, cessation, and conscious modification of mastication are dictated by the cerebral cortex, the sustenance of the ongoing, repetitive chewing cycle is remarkably autonomous [9,10]. Once initiated, mastication transitions smoothly into an automatic, stereotypical motor program driven by an intricate network of interneurons residing in the brainstem, designated as the masticatory CPG. The masticatory CPG represents a highly specialized neural circuit capable of generating organized, rhythmic, and alternating motor outputs to antagonistic jaw muscles completely in the absence of periodic external sensory or descending inputs [9,10]. Rhythmogenesis emerges not only from intrinsic membrane properties but also from network-level interactions, including reciprocal inhibition and synaptic coupling. Similar ionic mechanisms have been described in other central pattern generators, such as the respiratory preBötzinger complex, suggesting a conserved rhythmogenic framework [11,12].

The fundamental challenge inherent to the masticatory CPG lies in its paradoxical requirement to balance rigid structural stability with highly dynamic flexibility. Unlike the respiratory CPG, which operates under relatively constant internal parameters, the masticatory CPG must continuously and instantaneously adjust its motor output based on the unpredictable physical properties of a food bolus [10,12]. As the bolus undergoes progressive fragmentation, its hardness, elasticity, and spatial position shift continuously. The CPG integrates these fluid peripheral sensory signals with descending cortical intents, dynamically shifting chewing velocity, cycle duration, and occlusal force vectoring.

At the cellular level, the intrinsic electrophysiological properties of CPG interneurons and trigeminal motoneurons are largely determined by the complement of ion channels expressed in their membranes [13]. In this review, the term “masticatory CPG” refers primarily to rhythmogenic and pattern-generating interneuronal networks located within the brainstem reticular formation. Trigeminal motoneurons are considered downstream output elements rather than core components of the CPG itself, although their intrinsic ion-channel properties play an important role in shaping the final expression of masticatory motor patterns. These ion channels do not merely act as binary transducers of synaptic inputs; instead, their voltage-dependent kinetics and intracellular second-messenger sensitivities allow them to generate complex autonomous subthreshold oscillations, plateau potentials, and bursts of action potentials [13,14]. However, direct evidence linking specific ion-channel subtypes to mastication in vivo remains limited. Understanding the precise molecular identity and biophysical properties of these channels is critical to resolving how the brain codes complex orofacial behaviors. This review integrates historical foundations with cutting-edge molecular, electrophysiological, and computational findings to map out how ion channels govern the central control of mastication. Because much of the current mechanistic framework has been inferred from other mammalian CPGs, direct evidence from masticatory CPG neurons is explicitly distinguished from extrapolated concepts throughout this review.

## 2. Hierarchical Neural Control of Mastication

The neural architecture regulating masticatory movements is organized in a distributed, hierarchical fashion across multiple axes of the neuraxis. Volitional control, motor pattern generation, reflex loops, and sensorimotor correction are integrated via a complex loop consisting of cortical, subcortical, brainstem, and peripheral elements. These findings highlight that mastication is governed by distributed neural circuits rather than a single localized center.

### 2.1. Cortical and Subcortical Command Structures

The primary engine for the voluntary orchestration of chewing is the cortical masticatory area (CMA), located within the lateral agranular frontal cortex and the anterior insular/opercular cortex [9,10]. Repetitive electrical or optogenetic stimulation of the CMA in animals induces highly coordinated, rhythmic jaw movements that mimic natural masticatory trajectories, driving sequential activation of jaw-opening and jaw-closing musculature [1,15,16]. The CMA projects to the brainstem reticular formation via corticobulbar pathways, where descending excitatory inputs activate rhythmogenic interneuronal circuits within the masticatory CPG, enabling the generation of coordinated rhythmic jaw movements [1,9,13].

Subcortical modulation is provided by the basal ganglia and the amygdaloid complex. The basal ganglia (specifically the striatum and substantia nigra) exert tonic GABAergic inhibition over motor-initiating centers. During feeding behavior, this inhibition is selectively lifted through the activation of the direct pathway, allowing the initiation of mastication [17]. Concurrently, the amygdala integrates metabolic status and emotional valency, linking the internal drive states of hunger or satiety to the activation of the masticatory apparatus [18].

### 2.2. The Brainstem CPG

The rhythm-generating network of the masticatory system is located entirely within the pons and medulla oblongata. Anatomically, the masticatory CPG is not confined to a single microscopically visible nucleus; rather, it is distributed across a structural continuum within the brainstem reticular formation. Accumulating evidence from transection, lesion, and localized microinjection studies indicates that key rhythmogenic interneuronal networks of the masticatory CPG are distributed within the peritrigeminal region, particularly the dorsomedial principal trigeminal sensory nucleus and the adjacent parvocellular reticular formation [13,19,20].

Recent activity-mapping and optogenetic studies further suggest that cortical and sensory inputs activate partially distinct components of the brainstem masticatory circuitry, with convergence occurring within the parvocellular reticular formation, supporting the concept that mastication emerges from a distributed network rather than a single rhythmogenic locus [16]. Recent advances in optogenetic approaches have enabled cell-type-specific interrogation of orofacial neural circuits, providing new opportunities to dissect the contribution of identified neuronal populations to mastication-related behaviors [21].

The functional organization of the masticatory CPG is commonly conceptualized as comprising two functionally distinct but highly interconnected modules: a rhythm generator, which determines the timing of the chewing cycle, and a pattern generator, which shapes the spatiotemporal activation of jaw muscles [10,13]. The rhythm generator establishes the fundamental frequency or cadence of the chewing cycle—typically 1.5 to 4.5 Hz across various mammalian species—and delivers this timing signal to the pattern generator [9,19]. The pattern generator then distributes the rhythmic drive into precisely timed, alternating commands directed to jaw-opening and jaw-closing motoneuron pools, a process shaped by the intrinsic membrane properties and ion-channel dynamics of interneurons and motoneurons [13,14].

This precise alternation is achieved via an intricate network of reciprocal inhibition [10,12,13]. Interneurons associated with the jaw-opening phase inhibit jaw-closing premotor circuits primarily through glycinergic and GABAergic neurotransmission. As this inhibition subsides at the end of the opening phase, jaw-closing premotor neurons become active, driving the coordinated activation of trigeminal motoneurons responsible for jaw closure [10,13,14]. Although trigeminal motoneurons are not generally regarded as constitutive elements of the rhythm-generating CPG network, their intrinsic membrane properties significantly shape motor pattern output and therefore contribute to the overall expression of masticatory rhythms.

### 2.3. The Trigeminal Motor Nucleus

The trigeminal motoneurons within the trigeminal motor nucleus, located in the dorsolateral pontine tegmentum, constitute the final common pathway through which central masticatory commands are conveyed to the jaw muscles [9,10,22]. The trigeminal motor nucleus exhibits a well-defined somatotopic organization, with distinct motoneuron pools corresponding to individual jaw muscles. Motoneurons innervating the anterior belly of the digastric muscle are located predominantly in the dorsomedial division, whereas those supplying the major jaw-closing muscles, including the masseter, temporalis, and medial pterygoid muscles, occupy the larger ventrolateral division [9,22]. Trigeminal motoneurons possess sophisticated intrinsic membrane properties, including delayed rectification and spike-frequency adaptation, which enable them to faithfully transform rhythmic synaptic inputs from the masticatory CPG into appropriately graded muscle activation [10,13,14].

### 2.4. Sensory Feedback and Pre-Programmed Reflex Loops

While the brainstem CPG can generate rhythmic output in the absence of sensory feedback, natural chewing requires continuous sensory modulation to prevent self-injury and optimize efficiency [10]. This modulation is achieved via two primary classes of intraoral peripheral receptors: Periodontal mechanoreceptors, embedded within the collagenous periodontal ligament, and muscle spindles, located in high density within the elevator muscles (masseter and temporalis).

A distinctive anatomical feature of the masticatory system is that the primary afferent cell bodies of jaw-muscle spindle afferents and a subset of low-threshold periodontal mechanoreceptor afferents are located within the mesencephalic trigeminal nucleus (MTN), rather than in peripheral sensory ganglia as in virtually all other somatic sensory systems [23]. This structure consists of a slender column of unipolar primary sensory neurons extending from the dorsal pons into the midbrain.

**Figure 1:**
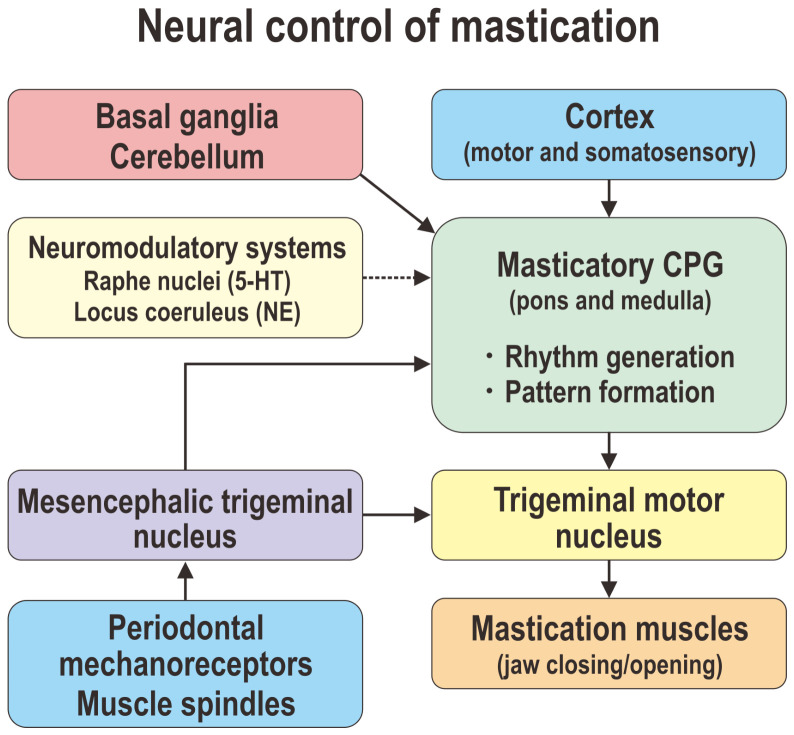
Hierarchical organization of the neural control of mastication. Volitional initiation of mastication originates in the CMA and associated sensorimotor cortical regions. Descending corticobulbar pathways activate the brainstem masticatory CPG, located primarily within the peritrigeminal region and parvocellular reticular formation. The rhythm generator establishes the basic chewing cadence, whereas the pattern generator organizes phase-specific activation of jaw-opening and jaw-closing motor pools. Trigeminal motoneurons serve as the final common pathway to the masticatory muscles. Continuous sensory feedback from periodontal mechanoreceptors, muscle spindles, and neurons of the MTN dynamically modulates ongoing chewing cycles. Basal ganglia, cerebellar, and limbic circuits provide additional modulatory control over movement initiation, force generation, and adaptive motor learning. Monoaminergic neuromodulatory inputs arising from the raphe nuclei (serotonin) and locus coeruleus (noradrenaline) regulate the excitability and rhythmic activity of the masticatory CPG. The cerebellum further contributes to masticatory control by optimizing movement accuracy, coordinating force production, and supporting adaptive sensorimotor learning during chewing.

The large pseudo-unipolar neurons of the mesencephalic trigeminal nucleus send peripheral branches to jaw-muscle spindles and central axons, including direct projections to trigeminal motoneurons, thereby contributing to the excitatory jaw-closing (jaw-jerk) reflex pathway. When the jaw is unexpectedly depressed or encounters a hard object, stretch-sensitive muscle spindle afferents in the masseter muscle rapidly increase their firing, and their MTN neurons monosynaptically excite trigeminal α-motoneurons, thereby recruiting additional jaw-closing motor units [10,22]. Concurrently, these sensory collaterals provide sensory input to interneuronal networks within the masticatory CPG, thereby resetting or prolonging specific phases of the chewing cycle to adapt to the obstacle [10,13]. In addition to mechanoreceptive feedback, mastication is influenced by multiple oral sensory modalities. Gustatory signals affect food acceptance and feeding motivation through interactions with brainstem and forebrain circuits. Thermal and tactile stimuli contribute to the evaluation of food properties and may modify chewing intensity and rhythm. Food texture, hardness, and viscosity strongly influence jaw-muscle activation patterns, chewing cycle duration, and bite force through sensorimotor integration mechanisms involving trigeminal sensory pathways. These multimodal sensory inputs allow mastication to adapt dynamically to changing food characteristics and behavioral contexts. The hierarchical organization of these distributed control systems is summarized in Figure 1. Having established the anatomical hierarchy of masticatory control, the following sections examine how intrinsic membrane properties enable rhythm generation within individual CPG neurons.

## 3. Biophysical Foundations of Neuronal Excitability

To understand how individual brainstem neurons transition into automated rhythm generators, one must examine the underlying biophysical principles that govern the neuronal plasma membrane [12,24,25]. The electrical behavior of CPG neurons is defined by the selective permeability of their lipid bilayers to major physiological ions: sodium, potassium, calcium, and chloride [26]. The baseline resting membrane potential is primarily set by the passive leakage of potassium ions through two-pore domain potassium channels and the electrogenic action of the sodium–potassium pump, creating a highly polarized state, with the membrane potential typically resting between −60 mV and −70 mV [24].

The generation and propagation of electrical signals are driven by the opening of voltage-gated ion channels in response to deviations from this resting potential. When the membrane potential changes, the total current crossing the lipid bilayer is determined by the combined behavior of the membrane capacitance and the individual ionic conductances [26]. Each type of channel possesses specific voltage-dependent parameters that dictate its activation and inactivation timing, creating a unique electrochemical driving force based on the internal and external concentration gradients of each ion. The rapid upstroke of an action potential is mediated by conventional voltage-gated sodium channels [24]. Upon depolarization past a critical threshold (typically −45 mV), the activation gates open rapidly, allowing a massive influx of sodium ions down their steep electrochemical gradient. Within milliseconds, the intrinsic inactivation gates close, terminating the inward current.

Repolarization and hyperpolarization are driven by an array of voltage-gated potassium channels [24]. Delayed rectifier potassium currents activate with a slight time delay relative to sodium influx, allowing potassium to exit the cell down its concentration gradient, bringing the membrane potential back toward resting levels. Transient, low-threshold A-type potassium currents activate upon depolarization from hyperpolarized potentials, acting as an electrical brake that slows subthreshold depolarization and regulates the precise timing between successive action potentials [27].

Crucially for motor pattern generation, CPG neurons express specialized ion channels that contribute to subthreshold membrane dynamics [14]. Although many ion channels contribute to neuronal excitability, this review focuses on conductances that have been implicated in rhythm generation or burst modulation within mammalian CPGs. These channels operate at membrane potentials well below the threshold for standard action potential generation. Through the complex interplay of three specific subthreshold conductances—the persistent sodium current, the hyperpolarization-activated cyclic nucleotide-gated current, and the low-threshold T-type calcium current—individual interneurons can generate complex autonomous subthreshold oscillations, plateau potentials, and bursts of action potentials [12,13,14]. Rhythmogenesis emerges not only from intrinsic membrane properties but also from network-level interactions, including reciprocal inhibition and synaptic coupling [12,13,14]. Similar ionic mechanisms have been described in other central pattern generators, such as the respiratory preBötzinger complex, suggesting a conserved rhythmogenic framework [11,12].

## 4. Ion-Channel Dynamics Within the Masticatory CPG

Rhythmic activity within the masticatory CPG emerges from the interaction between intrinsic membrane properties of rhythmogenic neurons and the synaptic connectivity of the underlying neural network [12,13,14]. The cellular pacemaking within the trigeminal sensory and reticular interneurons relies on a delicate balance of specialized voltage-gated and ligand-gated ion channels. Most ion-channel mechanisms discussed in this section derive from studies of rhythmogenic and premotor interneurons, which are generally regarded as the core cellular elements of the masticatory CPG. Nevertheless, many of the same conductances are also expressed in trigeminal motoneurons, where they influence neuronal excitability, spike-frequency adaptation, and the transformation of CPG output into coordinated muscle activation patterns. However, direct evidence linking specific ion-channel subtypes to mastication in vivo remains limited. Recent evidence further indicates that astrocytes actively participate in masticatory rhythmogenesis through gliotransmitter-mediated modulation of neuronal excitability, suggesting that rhythm generation emerges from neuron–glia interactions rather than exclusively neuronal mechanisms [28]. The principal ion channels and receptors currently implicated in masticatory rhythm generation are summarized in Table 1.

### 4.1. The Persistent Sodium Current: The Engine of Burst Initiation

Whereas the classical transient sodium current (I_NaT_) inactivates rapidly within a few milliseconds following membrane depolarization, a small non-inactivating component persists during sustained depolarization. This persistent sodium current (I_NaP_) is thought to arise from a subpopulation of voltage-gated sodium channels exhibiting incomplete or slow inactivation, thereby providing a sustained inward depolarizing drive that enhances neuronal excitability [24,29]. The I_NaP_ is strongly enriched in the axon initial segment, where voltage-gated sodium channels such as Nav1.6 and Nav1.2 are densely clustered [31,37]. It activates at subthreshold membrane potentials near the range of approximately −65 to −60 mV, below the threshold for transient sodium current activation, thereby facilitating spike initiation and sustained depolarization in CPG interneurons [24,29,31].

Because the I_NaP_ is a depolarizing inward current that increases with membrane depolarization over the subthreshold range, it can generate a regenerative amplification of excitatory drive. When a neuron is slightly depolarized from its resting potential, activation of this current further enhances sodium influx, leading to additional depolarization and the emergence of a positive feedback loop in membrane excitability. This property contributes to the generation of plateau potentials—prolonged depolarized membrane states associated with sustained or high-frequency burst firing in central pattern generator neurons [11,12,13,29,38].

In computational models and in vitro preparations of brainstem rhythm-generating circuits, including preBötzinger complex models, reduction of I_NaP_—either pharmacologically using agents such as riluzole or indirectly through partial sodium channel block—has been shown to significantly disrupt rhythmic bursting activity in interneuronal networks. These findings suggest that I_NaP_ appears to provide an important inward depolarizing drive that supports burst initiation and the maintenance of rhythmic oscillatory behavior [13,29,39,40,41]. Although this mechanism is well established in several mammalian CPGs, direct electrophysiological confirmation in identified masticatory CPG neurons remains limited.

### 4.2. T-Type Calcium Channels: Drivers of Low-Threshold Bursting

The low-threshold, transient T-type calcium current, mediated by Cav3 family channels (Cav3.1, Cav3.2, and Cav3.3), plays a key role in post-inhibitory rebound excitation and burst initiation in central pattern generator neurons, thereby contributing to phase transitions within rhythmic motor programs such as mastication [32]. Unlike high-voltage-activated calcium channels (L-, N-, and P/Q-types) that require massive depolarization to open, T-type channels activate near the resting membrane potential (approximately −60 mV). T-type calcium channels exhibit strong voltage-dependent steady-state inactivation at membrane potentials near the resting level, resulting in limited channel availability under depolarized conditions. Recovery from inactivation requires prior membrane hyperpolarization, which shifts channels into a de-inactivated state and renders them available for subsequent activation. This property enables T-type channels to function as critical determinants of post-inhibitory excitability and rebound firing in neuronal networks [32,42,43].

This property underlies rebound bursting, a fundamental mechanism in central pattern generator networks. During the jaw-opening phase, masticatory interneurons receive strong glycinergic or GABAergic inhibition from antagonistic premotor populations, resulting in membrane hyperpolarization that relieves the voltage-dependent inactivation of low-threshold conductances. Upon release from inhibition, these conductances support post-inhibitory rebound excitation and burst generation, contributing to the alternation of jaw-opening and jaw-closing motor activity [12,13,32,42]. Upon termination of inhibitory synaptic input, membrane depolarization from a hyperpolarized state activates T-type calcium channels, generating a transient low-threshold calcium spike (LTS). This LTS provides a depolarizing plateau that triggers a burst of action potentials [32,42,44,45]. Although direct evidence in identified masticatory rhythm-generating neurons remains limited, similar mechanisms have been consistently demonstrated in several mammalian CPGs. This mechanism contributes to the alternating activation of jaw-opening and jaw-closing motor pools via reciprocal post-inhibitory rebound.

### 4.3. Hyperpolarization-Activated Cyclic Nucleotide-Gated (HCN) Channels

The hyperpolarization-activated cation current (Ih), mediated by hyperpolarization-activated cyclic nucleotide-gated (HCN) channels, is expressed in trigeminal brainstem neurons, with HCN1 and HCN2 subunits being the predominant isoforms reported in this system. This current contributes to membrane depolarization from hyperpolarized potentials and plays a role in regulating neuronal excitability and rhythmic activity in brainstem circuits [33,46,47]. HCN channels possess a highly unconventional biophysical profile: they are closed at depolarized membrane potentials and open slowly when the membrane potential hyperpolarizes below −65 mV.

Following the termination of a burst of action potentials, CPG interneurons typically enter a hyperpolarized membrane potential state. This hyperpolarization activates HCN channels, generating an inward current (Ih) carried primarily by Na^+^ and K^+^ ions. This slowly activating depolarizing current contributes to membrane potential stabilization and influences the timing of interburst intervals, thereby participating in the regulation of rhythmic activity in central pattern generator networks [12,33,47,48]. This inward current slowly drives the membrane potential toward depolarization, contributing to a slow depolarizing ramp known as the pacemaker potential. The slope of this pacemaker potential contributes to the precise length of the interburst interval; steeper slopes allow the membrane potential to reach activation threshold sooner for the persistent sodium and T-type calcium channels, accelerating the overall masticatory cycle frequency.

Furthermore, HCN channel gating is modulated by intracellular cyclic adenosine monophosphate (cAMP). Intracellular binding of cAMP shifts the activation curve of HCN channels toward more positive potentials, allowing them to open faster and at shallower hyperpolarizations. This neuromodulatory pathway allows monoaminergic systems, including noradrenergic and serotonergic projections originating from brainstem nuclei, to dynamically regulate the excitability of masticatory network circuits. During changes in behavioral state or increased mechanical demands during chewing, these modulatory inputs may adjust the rhythm and pattern of chewing by altering the intrinsic and synaptic properties of brainstem central pattern generator circuits [10,13]. Recent optogenetic activation of serotonergic neurons has been shown to alter masticatory motor outputs, supporting the concept that neuromodulatory pathways dynamically regulate brainstem chewing circuits [49].

### 4.4. Calcium-Activated Potassium Channels: Mediators of Burst Termination

To sustain a stable rhythmic output, burst-generating neurons in CPG circuits engage termination mechanisms that counterbalance depolarizing inward currents. A key component of this process is mediated by calcium-activated potassium (KCa) channels, which translate intracellular calcium signals into outward potassium currents that promote membrane repolarization and burst termination.

Calcium-activated potassium channels are broadly classified into two major functional groups: large-conductance (BK) channels, which are activated synergistically by membrane depolarization and increases in intracellular Ca^2+^, and small-conductance (SK) channels, which are activated primarily by Ca^2+^ binding via calmodulin in a voltage-independent manner [34,50].

During burst activity in CPG neurons, depolarization driven by I_NaP_ and low-threshold calcium currents promotes Ca^2+^ influx through voltage-gated calcium channels. The resulting increase in intracellular Ca^2+^ in microdomains near the plasma membrane activates SK and BK channels. SK channels are particularly sensitive to intracellular Ca^2+^ elevations via calmodulin binding, whereas BK channels integrate both Ca^2+^ concentration and membrane voltage to regulate their open probability [34,51].

Activation of K_Ca_ channels generates an outward potassium current that opposes ongoing depolarizing currents, thereby contributing to membrane repolarization and termination of burst firing. BK channels primarily shape rapid spike repolarization, whereas SK channels contribute predominantly to burst termination and the generation of afterhyperpolarization. This hyperpolarizing influence contributes to the afterhyperpolarization (AHP) phase, which regulates interburst intervals and stabilizes rhythmic activity in CPG networks [50]. Direct evidence for this mechanism in identified masticatory rhythmogenic neurons remains incomplete, although related mechanisms have been demonstrated in trigeminal neurons and other mammalian CPGs.

### 4.5. NMDA Receptors: Synaptic Conductances and Signaling Mechanisms

Beyond intrinsic voltage-gated conductances, masticatory CPG networks also depend on synaptic conductances mediated by ionotropic glutamate receptors. Among these, N-methyl-D-aspartate (NMDA) receptors contribute to sustained excitatory synaptic drive in brainstem rhythm-generating circuits [10,52]. NMDA receptors exhibit a characteristic voltage-dependent magnesium (Mg^2+^) block. At hyperpolarized membrane potentials, Mg^2+^ ions occlude the channel pore despite glutamate binding. Depolarization of the postsynaptic membrane relieves this voltage-dependent block, allowing a sustained inward flux of Na^+^ and Ca^2+^ ions [53,54].

AMPA receptors mediate the initial fast excitatory synaptic transmission, whereas NMDA receptors prolong depolarization after relief of the voltage-dependent Mg^2+^ block. This voltage-dependent property enables NMDA receptors to contribute to nonlinear synaptic integration and sustained depolarizing drive during network activity. The contribution of NMDA receptors to synaptic integration, plasticity, and network regulation has been comprehensively reviewed in recent studies [55,56].

**Figure 2:**
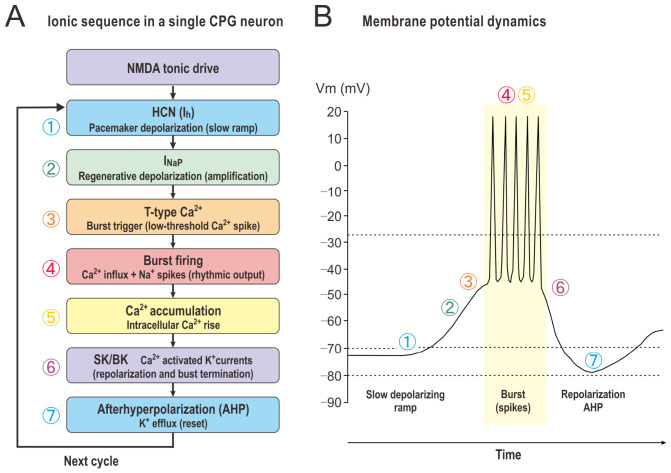
Proposed ion-channel interactions underlying rhythmic burst generation in masticatory CPG neurons. Following burst termination, membrane hyperpolarization activates HCN channels, generating an inward depolarizing current (Ih). This pacemaker depolarization progressively recruits INaP and low-threshold T-type calcium channels, producing regenerative depolarization and burst initiation. During burst firing, calcium influx accumulates and activates calcium-dependent potassium channels (SK/BK), which generate outward potassium currents that terminate the burst and restore membrane hyperpolarization. NMDA receptors provide sustained excitatory synaptic drive and facilitate network synchronization.

Within brainstem masticatory circuits, descending corticobulbar glutamatergic inputs from cortical masticatory-related regions are thought to activate both AMPA- and NMDA-type glutamate receptors in reticular formation and trigeminal premotor networks. This combined glutamatergic drive is thought to contribute to the maintenance of rhythmic activity in masticatory CPG networks and the generation of coordinated jaw movements [10,13,14]. A conceptual model of the proposed interactions among these ion channels is shown in Figure 2.

This model integrates available evidence from trigeminal neurons together with mechanistic concepts derived from other mammalian rhythm-generating networks. The figure represents a conceptual framework rather than a direct experimental reconstruction of membrane potential dynamics in identified masticatory CPG neurons. The temporal sequence shown is simplified for illustrative purposes, as individual conductances overlap substantially during physiological rhythm generation. Accordingly, the sequence is intended to emphasize the predominant contribution of each conductance rather than imply strictly sequential activation.

The ion-channel mechanisms proposed for the masticatory CPG share several features with other biological rhythm generators. Similar contributions of persistent sodium currents, T-type calcium channels, and HCN channels have been implicated in respiratory rhythm generation within the pre-Bötzinger complex and in thalamic oscillatory networks [11,12,32,33,39,40,41,42,43,47,48]. In addition, HCN-mediated pacemaker depolarization represents a common feature shared with cardiac sinoatrial node pacemaker cells [33,47]. These observations suggest that the masticatory CPG may utilize conserved rhythmogenic principles while retaining specialized adaptations for sensorimotor integration and food-dependent behavioral flexibility.

Taken together, these conductances may act sequentially and cooperatively, forming a cyclic rhythmogenic mechanism in which HCN channels contribute to pacemaker depolarization, persistent sodium and T-type calcium channels promote burst initiation, and calcium-activated potassium channels terminate bursting activity (Figure 2).

## 5. Peripheral Translation of Rhythmic Motor Commands

A detailed discussion of skeletal muscle excitation–contraction coupling is beyond the scope of this review. Briefly, rhythmic motor commands generated by the masticatory CPG are conveyed through trigeminal motoneurons to the jaw muscles, where neuromuscular transmission and excitation–contraction coupling convert neural activity into force production [57,58,59,60]. Through this process, centrally generated rhythmic activity is translated into coordinated jaw-opening and jaw-closing movements required for mastication [57,58]. Thus, ion-channel activity throughout the neuromuscular pathway ultimately ensures that brainstem-generated masticatory rhythms are expressed as coordinated motor behavior.

## 6. Clinical Implications and Channelopathies

Given the critical reliance of the masticatory apparatus on ion-channel dynamics, alterations in channel expression or function may contribute to orofacial motor dysfunction and broader neurological disorders [28,61]. Such clinical manifestations span localized masticatory dysfunctions as well as systemic neurodegenerative conditions [61], highlighting the potential translational relevance of ion channels as therapeutic targets in orofacial motor disorders.

### 6.1. Orofacial Disorders

Genetic, epigenetic, or activity-dependent modifications of ion channels in CPG networks and motoneurons may contribute to orofacial motor dysfunction. Awake and sleep bruxism—characterized by repetitive or sustained jaw muscle activity—is increasingly considered a central motor dysregulation of the masticatory system rather than a purely peripheral occlusal disorder [62].

Alterations in intrinsic excitability mechanisms, such as changes in I_NaP_ or small-conductance potassium channel activity in trigeminal motoneurons, have been proposed to contribute to increased motor excitability and prolonged muscle activation patterns [63,64]. Such changes may reduce afterhyperpolarization-mediated inhibition and bias the system toward sustained jaw-closing activity, potentially overriding normal reciprocal inhibition between jaw-opening and jaw-closing circuits [19,63].

Temporomandibular disorders (TMD), characterized by chronic pain and dysfunction of the temporomandibular joint, are associated with both peripheral and central sensitization mechanisms involving altered ion-channel function [65,66]. Prolonged mechanical stress and inflammatory signaling within the TMJ can induce the release of cytokines such as tumor necrosis factor-alpha and interleukin-1 beta [67], which activate intracellular kinase pathways that modulate nociceptive excitability. These processes can sensitize transient receptor potential vanilloid 1 (TRPV1) channels and voltage-gated sodium channels, contributing to peripheral sensitization and chronic myofascial pain [66,68].

### 6.2. Neurodegenerative Diseases

Impairments in masticatory function are frequently observed in several major neurodegenerative and motor system disorders. In amyotrophic lateral sclerosis (ALS), including bulbar-onset variants, degeneration of motoneurons in the trigeminal motor nucleus and nucleus ambiguus leads to progressive impairment of orofacial motor function [69]. Early stages of the disease may be accompanied by fasciculations and altered jaw muscle excitability, which have been associated with abnormalities in potassium channel function and I_NaP_. These channel abnormalities have been proposed to contribute to motoneuron hyperexcitability [63,70,71,72]. These electrophysiological alterations may further promote calcium dysregulation and excitotoxic mechanisms that accelerate motoneuron degeneration [73].

Masticatory dysfunction is also common in Parkinson’s disease (PD), where patients frequently exhibit jaw rigidity, bradykinesia of chewing cycles, impaired bolus formation, and an increased risk of dysphagia [74,75]. These abnormalities are primarily attributed to degeneration of dopaminergic pathways within basal ganglia-brainstem motor control circuits. Loss of dopaminergic modulation has been suggested to alter the excitability of distributed motor networks, including ion-channel-dependent mechanisms involving HCN channels and NMDA receptor-mediated signaling, thereby reducing the flexibility and adaptability of motor pattern generation required for normal mastication [76].

Beyond classical motor disorders, accumulating evidence also suggests a relationship between long-term masticatory dysfunction and cognitive decline. Accumulating epidemiological and experimental evidence indicates a significant association between long-term masticatory impairment, including tooth loss and chronic consumption of soft diets, and an increased risk of cognitive decline, dementia, and Alzheimer’s disease [77,78]. One proposed mechanism underlying this relationship is the mastication–cognition axis, whereby rhythmic activation of periodontal mechanoreceptors and mesencephalic trigeminal proprioceptors provides continuous sensory input to brainstem and cortical networks involved in arousal, attention, and cognitive processing [79,80,81]. Through these neural pathways, masticatory activity may contribute to the maintenance of cortical activation and cerebral perfusion, particularly in regions associated with learning and memory [7].

Experimental studies further suggest that chronic reductions in masticatory activity can induce structural and functional alterations within the hippocampus and related cognitive centers. Decreased masticatory input has been associated with reduced c-Fos expression, diminished dendritic spine density, impaired synaptic plasticity, and increased tau phosphorylation, changes that are linked to cognitive dysfunction and neurodegenerative processes [3,82,83]. These observations support the concept that preservation of adequate masticatory function through dental rehabilitation, prosthodontic treatment, or oral functional training may help attenuate age-related cognitive decline and structural brain atrophy [80].

### 6.3. Therapeutic Perspectives

Given the evidence discussed above, ion channels have emerged as attractive therapeutic targets for disorders involving abnormal motor pattern generation and neurodegeneration [84]. Potential approaches include modulation of I_NaP_ using agents such as riluzole to reduce pathological neuronal hyperexcitability in conditions associated with central pattern generator dysfunction and motoneuron hyperactivity [85]. Additional strategies may involve positive modulation of small-conductance calcium-activated potassium (SK) channels, which can enhance afterhyperpolarization, promote burst termination, and improve motor pattern stability [63,86]. Furthermore, modulation of hyperpolarization-activated cyclic nucleotide-gated (HCN) channels may provide a means of regulating rhythmic neuronal activity and stabilizing oscillatory network behavior in disorders characterized by abnormal motor rhythms [87,88]. These therapeutic strategies remain largely speculative for mastication-specific disorders and require experimental validation.

## 7. Limitations of Current Evidence

Despite substantial progress in understanding the neural control of mastication, several important limitations remain. First, direct electrophysiological evidence linking specific ion-channel subtypes to rhythm generation in identified masticatory CPG neurons remains relatively scarce. Many mechanistic concepts discussed in this review, particularly those concerning I_NaP_, HCN channels, and T-type calcium channels, are supported by findings obtained from other rhythmogenic networks such as the respiratory preBötzinger complex and spinal locomotor CPGs. Although these systems likely share common rhythmogenic principles, direct extrapolation to the masticatory system should be interpreted cautiously.

Second, the precise anatomical boundaries and cellular composition of the masticatory CPG remain incompletely defined. Recent studies indicate that the peritrigeminal region and parvocellular reticular formation contain multiple functionally distinct neuronal populations involved in sensory integration, rhythmogenesis, and premotor processing [13,16,19,20]. These findings suggest that masticatory rhythm generation emerges from distributed network interactions rather than from a single anatomically localized kernel.

Third, most available studies have been performed in rodents, and species-specific differences in the organization of human chewing networks remain poorly characterized. Direct translational evidence linking ion-channel dysfunction to human masticatory disorders is therefore limited.

Finally, emerging technologies including optogenetics, chemogenetics, calcium imaging, connectomics, and single-cell transcriptomics have only recently begun to be applied to masticatory circuits. Combining these technologies with in vivo behavioral analyses will be particularly important for validating the proposed conceptual framework presented in this review. Integration of these approaches will be essential for establishing causal relationships between molecular ion-channel expression, cellular excitability, and behavioral chewing patterns.

## 8. Conclusions and Future Perspectives

The neural control of mastication represents an elegant biological solution to a complex biomechanical challenge. By embedding rhythmic motor pattern generation within a distributed brainstem CPG, the mammalian nervous system helps to streamline the control of routine chewing at higher cortical levels, while preserving the capacity for voluntary, top–down modulation [10,89].

As discussed in this review, the functional flexibility of this network is shaped by a diverse array of ion channels expressed in brainstem interneurons, trigeminal motoneurons, and peripheral muscle fibers [90]. The rhythmic pattern of mastication emerges from the biophysical interaction between inward depolarizing currents and opposing hyperpolarizing conductances, which together regulate neuronal excitability and network timing.

However, direct evidence linking specific ion-channel subtypes to the generation and modulation of masticatory rhythms in vivo remains limited. Future studies integrating electrophysiology, imaging, molecular genetics, connectomics, and computational neuroscience will be essential to establish causal relationships between specific ion channels and rhythm generation in the mammalian masticatory CPG.

A more complete characterization of these channel dynamics is expected to deepen our understanding of mammalian sensorimotor organization and may inform the development of targeted therapeutic strategies for orofacial motor dysfunction, chronic craniofacial pain, and age-related decline in motor control. Ultimately, a deeper understanding of ion-channel dynamics within the masticatory CPG will provide an essential framework for elucidating the neural basis of rhythmic orofacial behaviors and their dysfunction in disease.

## Figures and Tables

**Table 1:** Key Ion Channels and Receptors in the Masticatory Central Pattern Generator.

Channel Type/Current	Primary Molecular Alpha Subunits	Primary Biophysical Role in Masticatory CPG	Evidence Level	Evidence Source	Key Reference
Persistent Sodium	Nav1.6, Nav1.2	Subthreshold regenerative depolarization; burst initiation and plateau maintenance.	Moderate	Indirect	[29,30,31]
T-type Calcium	Cav3.2, Cav3.3	Low-threshold calcium spikes; transitions neurons from tonic to burst firing modes via post-inhibitory rebound.	Moderate	Indirect	[32]
HCN/Pacemaker Current	HCN1, HCN2	Hyperpolarization-activated inward current; contributes to rhythm generation, interburst interval, and pacemaking cadence.	Limited	Indirect	[33]
Calcium-Activated Potassium	KCa1.1 (BK), KCa2.2 (SK)	Intracellular calcium sensory feedback; drives burst termination and phase transitions.	Moderate	Partial direct	[34,35]
NMDA Receptor Current	GluN1, GluN2A, GluN2B	Voltage-dependent magnesium block relief; provides non-linear sustained excitatory drive, coincidence detection, and synaptic plasticity.	Moderate	Partial direct	[36]

Evidence Level reflects confidence in the proposed physiological role of each conductance in masticatory rhythm generation. Moderate indicates substantial but incomplete experimental support, whereas Limited indicates primarily indirect or inferential evidence. Evidence Source refers to the origin of the supporting data. Direct indicates experiments performed within the masticatory CPG itself, Partial Direct indicates combined evidence from masticatory and other mammalian CPGs, and Indirect indicates evidence inferred predominantly from non-masticatory rhythm-generating networks.

## Data Availability

No new data were created or analyzed in this study. Data sharing is not applicable to this article.
